# Recognition of Lynch Syndrome Amongst Newly Diagnosed Colorectal Cancers at St. Paul's Hospital

**DOI:** 10.1155/2017/9625638

**Published:** 2017-07-02

**Authors:** Steven Pi, Estello Nap-Hill, Jennifer Telford, Robert Enns

**Affiliations:** ^1^Department of Medicine, University of British Columbia Faculty of Medicine, Vancouver, BC, Canada; ^2^Pacific Gastroenterology Associates, Vancouver, BC, Canada; ^3^Department of Gastroenterology, Division of Gastroenterology, St. Paul's Hospital, Vancouver, BC, Canada

## Abstract

**Background:**

Lynch Syndrome (LS) is the most common cause of inherited colorectal cancer (CRC). In British Columbia, most centres still use clinical criteria (Amsterdam II, Revised Bethesda, or the BC Cancer Agency's criteria) to determine who should undergo further first-line testing in the form of microsatellite instability or immunohistochemistry staining. Given the limitations with this strategy, LS is thought to be underrecognized.

**Objective:**

To investigate whether LS is truly underrecognized when compared to the reported prevalence.

**Methods:**

A retrospective chart review of all CRC cases diagnosed at St. Paul's Hospital from 2010 to 2013 was conducted.

**Results:**

246 patients met inclusion criteria. 76% (83/109) with a family history of malignancy were unable to recall the specific malignancy or age of diagnosis. 18% (43/235) were only asked about a history of gastrointestinal related malignancy and 26% (65/246) met at least one of the three criteria but only 21% (13/63) received further investigation. Only 1.6% (4/246) had LS compared to the reported prevalence of 2–5% of all CRC cases.

**Conclusion:**

This data supports our hypothesis that LS is underrecognized. Issues at the patient, physician, and systems level need to be evaluated to determine where the limitations preventing appropriate testing are occurring.

## 1. Introduction

Lynch Syndrome is the most common cause of inherited colorectal cancer (CRC), estimated to account for 2–5% of all CRC cases [1]. This autosomal dominant disorder is the result of a loss-of-function mutation in one of the DNA mismatch repair genes (*MLH1*,* MSH2*,* MSH6*, or* PMS2*). The presence of these mutations identifies the syndrome. Consequently, the inability to repair mismatched DNA ultimately leads to an increased risk of both colonic and extracolonic malignancies [1]. Depending on which gene is affected, patients may have an increased risk of endometrial, ovarian, upper urologic tract, gastric, small bowel, biliary/pancreatic, brain, and/or sebaceous gland malignancies. Identification of patients with Lynch Syndrome is essential because intensive cancer screening and prophylactic surgery have been shown to reduce incidence and mortality of both colonic [2] and extracolonic malignancies [3]. In addition, early identification permits directed genetic counselling for relevant family members.

Numerous strategies exist to identify patients at risk for Lynch Syndrome. These include clinical criteria such as the Amsterdam II or Revised Bethesda Guidelines (Table 1), prediction models such as PREMM or MMRPredict, or tumour testing in the form of microsatellite instability (MSI) or immunohistochemistry (IHC) staining. In British Columbia, most centres will utilize the clinical criteria strategy to identify which patients should undergo further tumour testing in the form of microsatellite instability testing or immunohistochemistry staining. Traditionally, these clinical criteria include the Amsterdam II criteria and Revised Bethesda Guidelines; however the Hereditary Cancer Program (HCP) under the BC Cancer Agency has also developed its own criteria (Table 1) which has been shown to have a higher sensitivity and positive predictive value when compared to the Amsterdam II and Revised Bethesda Guidelines [4]. Patients referred to the HCP receive an appointment with an oncologist and geneticist and for further diagnostic testing such as MSI testing, IHC staining, and germline mutation testing, if necessary [5]. If these tests ultimately reveal a genetic mutation in the Lynch Syndrome associated genes then a diagnosis is made. Despite its ongoing use, a number of limitations exist for utilizing the clinical criteria approach to identify those who should undergo further testing for Lynch Syndrome. First, the patient must be aware of the exact age of diagnosis for any malignancy in their first- or second-degree relatives. Second, the consulting physician must complete a detailed past medical and family history of all malignancies in their first- and second-degree relatives. Third, the consulting physician must be aware of the above-mentioned criteria and when a referral to the Cancer Agency for further investigation would be appropriate. If any of these factors are missing, a diagnosis of Lynch Syndrome could be missed.

Due to these limitations, Lynch Syndrome is thought to be underrecognized at centres in British Columbia, such as St. Paul's Hospital (SPH), which utilize the clinical criteria approach. Of note, SPH is a major referral centre for colorectal cancers for the Province of British Columbia. This could lead to significant consequences, as a missed diagnosis could potentially lead to further malignancy that may have otherwise been preventable in either the patient or their family. The purpose of this study is to investigate whether Lynch Syndrome is truly underrecognized when compared to the reported prevalence and, if so, identifying what factors may be contributing to this.

## 2. Materials and Methods

### 2.1. Database

Patients were initially extracted using the St. Paul's Hospital Department of Pathology's database. A search for the string “adenocarcinoma” was run on all rectal, sigmoid, descending, transverse, ascending, and cecal biopsies between January 1, 2010, and December 31, 2013. These patients were then subsequently identified using the Pacific Gastroenterology Associates Electronic Medical Record to obtain further clinical information as outlined below.

### 2.2. Inclusion/Exclusion Criteria

Included in this study were patients referred to and seen by the St. Paul's Hospital gastroenterologists that had a pathologic diagnosis of CRC on endoscopic or surgical biopsy between Jan 1, 2010, and Dec 31, 2013. Our population of interest was nonurgent patients in the outpatient setting, as we assume that this would be the ideal setting for the consulting physician to complete a thorough family history and give consideration to investigating for Lynch Syndrome. Of note is that St. Paul's Hospital is a major referral centre for colorectal cancers for the Province of British Columbia.

Excluded in this study were patients seen in the Emergency Department, inpatients at St. Paul's Hospital, or patients in whom an urgent assessment was indicated (e.g., radiographic evidence suggestive of colorectal cancer or palpable rectal mass). Similarly, patients with known colorectal cancer who were referred to gastroenterology solely for a procedure (e.g., endoscopic ultrasound for staging of a known colorectal cancer) were excluded as these patients would also be less likely to receive a thorough family history. Patients with known IBD were excluded as the pathologic findings on their colon cancer could potentially confound the high-risk pathologic findings in the Revised Bethesda Criteria (i.e., Crohn's-like lymphocytic reaction). Patients were also excluded if they were enrolled under the Colon Check Pilot (CCP), the pilot program of the current British Columbia Colon Screening Program, as their screening and management were protocoled differently from the standard of care during that period of time.

### 2.3. Ethics

Providence Health Care Institutional Approval for ethics was obtained on July 7, 2016, REB number: H16-00881.

### 2.4. Data Extraction

Patients meeting inclusion criteria had the following data extracted: (1) full name; (2) year of birth; (3) sex; (4) provincial health number; (5) age of diagnosis of colon cancer; (6) indication for colonoscopy; (7) pathology report of colorectal cancer, including staging; (8) location of colorectal cancer on diagnostic and subsequent endoscopies or surgeries; (9) past medical history of previous cancers and, if so, which type and age of the respective diagnosis; (10) family history of previous cancers and, if so, which relatives, type, and age of respective diagnosis; (11) IHC/MSI results for patients/family members.

### 2.5. Data Analysis

**Figure 1:**
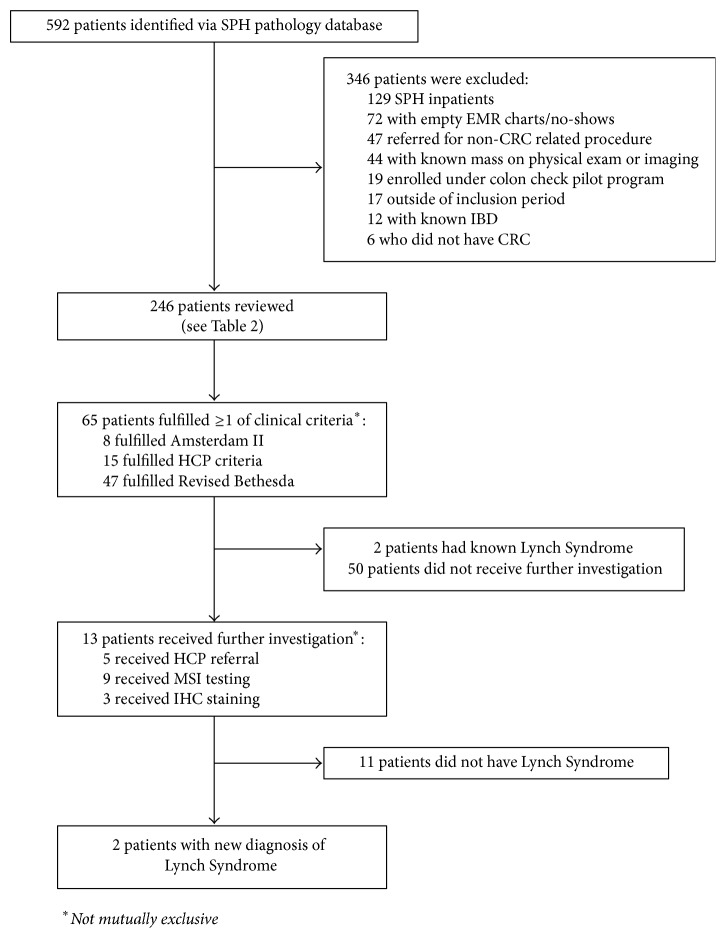
Flow chart of study and findings.

Each patient had their extracted data analyzed to see if any of the Amsterdam II, Revised Bethesda, or Hereditary Cancer Program criteria were met (Figure 1). Those who met any of the criteria were then assessed to see if they had received any form of further investigation for Lynch Syndrome in the form of either a referral to the Hereditary Cancer Program, MSI testing, or IHC staining. Those with positive results yielding a diagnosis of Lynch Syndrome were then used to calculate the proportion of Lynch Syndrome amongst newly diagnosed colorectal cancers at St. Paul's Hospital. This proportion was then compared to the reported baseline prevalence of 2–5% (BCCA 2012) to determine whether or not Lynch Syndrome is underrecognized. Two-tailed *t*-tests and chi-square testing were used to calculate statistical significance between the population who received further investigation and all patients included in the study.

### 2.6. Confidentiality

Data was stored on an encrypted password protected Excel 2012 spreadsheet. Each subject was assigned a unique identifier. Data was extracted respective to each unique identifier with no identifying information on the data spreadsheet.

## 3. Results

### 3.1. Excluded Patients

592 patients were identified after searching the pathology database (Figure 1) with the following patients excluded: 129 patients were inpatients or seen in the Emergency Department; 72 patients were not seen by the gastroenterologists (with empty EMR charts, not showing up for consultation, etc.); 47 patients with CRC were referred for reasons other than colonoscopy, for example, staging of a known rectal malignancy via endoscopic ultrasound. 44 patients were seen for highly suspected malignancy, for example, colonic mass on imaging or a suspicious rectal mass on exam. 19 patients were under the CCP; 17 patients were seen outside the period of inclusion; 12 patients had known IBD; 6 patients did not have CRC.

### 3.2. Patient Recollection of Family History of Malignancy

There were a total of 246 patients who met the inclusion criteria for this study (Table 2). Four patients had an incompletely documented initial consult; however information about these patients was obtainable through subsequent progress notes and was thus included in the study. 44% (109 of 246 patients) reported a positive family history for any malignancy. Of these, 76% (83 of 109 patients) were unable to recall either the specific malignancy or their respective age of diagnosis.

### 3.3. Completion of Family History by Physician

Of the 246 patients diagnosed with colorectal cancer, 96% (235 of 246 patients) had a family history available in their electronic chart. Of these, 46% (109 of 235 patients) had a family history of any malignancy, 35% (83 of 235 patients) had no family history of any malignancy, and 18% (43 of 235 patients) had a documentation of some equivalency of “no family history of gastrointestinal malignancy.”

### 3.4. Pursuing of Further Investigation by Physician

26% (65 of 246 patients) fulfilled at least one of the Amsterdam II (8 patients), Revised Bethesda (47 patients), or the HCP Criteria (15 patients). Two patients had known Lynch Syndrome. Of those without a known diagnosis, 21% (13 of 63 patients) went on to receive further investigation. Five patients were referred to the HCP, 9 received MSI testing, and 3 received IHC staining (not mutually exclusive). Patients who received further investigation had a statistically significant younger age, past medical history of malignancy, and family history of malignancy when compared to our baseline population (Table 3).

### 3.5. Diagnosis of Lynch Syndrome

Of the 246 patients diagnosed with colorectal cancer, 1.6% (4 patients) of these patients had a diagnosis of Lynch Syndrome. Two of these 4 patients had a previously known diagnosis of Lynch Syndrome, meaning that 0.8% (2 of 246 patients) were found to have a new diagnosis of Lynch Syndrome.

## 4. Discussion

Given that only 1.6% (4 of 246) of our population were diagnosed with Lynch Syndrome, our data suggests that Lynch Syndrome is indeed underrecognized when compared to the reported prevalence of 2–5% of all colorectal cancers. Our figure of 1.6% represents a population that reflects an ideal outpatient setting, where we assume that the physician (tertiary care gastroenterologist) would have the most time to complete a thorough family history and give consideration to investigating for Lynch Syndrome. Thus, our figure of 1.6% can even be considered as an overestimation of the true proportion of Lynch Syndrome amongst* all* newly diagnosed colorectal cancers at St. Paul's Hospital between 2010 and 2013. Even more concerning is the fact that St. Paul's is considered to be the major referral centre for complicated colorectal management in the Province of BC.

A number of contributing factors have emerged from this study. First, the majority of patients seem to have poor recognition of the exact type of malignancy and age of diagnosis for their family members, as 76% (83 of 109) of patients with a positive family history were unable to provide a history that was sufficient enough to be applied to any of the criteria. The exact type of malignancy can often be challenging for patients who may have difficulty discerning between various anatomical sites as well as conceptualizing primary versus metastatic disease. For example, misinterpreting a cervical cancer as an endometrial cancer can be misleading, as the latter is a Lynch Syndrome associated malignancy whereas the former is not. Another example would be a family member with metastatic disease to the liver from a colorectal cancer being misconstrued as a “liver cancer” which could mislead the physician into missing a family history of colorectal cancer. Furthermore, it is our experience that patients seem to have better recollection of their family member's age of death, as opposed to age of diagnosis. Overall, poor recollection of either the type of malignancy or its age of diagnosis inhibits the utility of the clinical criteria approach to identify patients at risk for Lynch Syndrome.

Second, our data suggests that although family history is discussed in the 96% (235 of 246 patients) of outpatient consultations, 18% (43 of 235 patients) of patients with a reportedly negative family history were only asked about a history of gastrointestinal malignancies. Given that Lynch Syndrome predisposes an individual to develop both colonic and extracolonic malignancy, a thorough family history exploring a history of any malignancy for all first-degree and second-degree relatives is warranted. It is entirely possible for a patient with unidentified Lynch Syndrome to have no family history of colorectal cancer but rather a significant history of endometrial cancer. Limiting the family history to solely gastrointestinal malignancies increases the risk of missing a diagnosis of Lynch Syndrome.

Third, only 21% (13 of 63 patients) of patients who met at least one of the criteria went on to receive further testing in the form of a HCP referral, MSI testing, or IHC staining. Our study suggests that patients with a younger age of diagnosis, past medical history of malignancy, and family history of malignancy are more likely to receive further investigation than those without. Given that this was a retrospective study, it is difficult to elicit from our data why some patients may not have received further investigation. However, we speculate that lack of recognition of the fulfillment of these criteria may be a factor. In addition, access to MSI testing or IHC staining is restricted and cannot be readily ordered by most physicians, including gastroenterologists, in British Columbia. This acts as an additional barrier to obtaining appropriate investigation for patients at high risk of Lynch Syndrome.

Overall, the underrecognition of Lynch Syndrome is multifactorial with issues at the patient, physician, and systems level. Although some factors are amenable to quality improvement intervention (e.g., encouraging physicians to ask about all malignancies in the family as opposed to just gastrointestinal related malignancy), some are not (e.g., poor patient recollection of the details of their family history). To deal with the fact that patients often have incomplete information and that physicians often do not (or cannot) obtain full family histories, the American Gastroenterology Association recommends universal MSI testing or IHC staining for all colorectal cancers [7]. Studies of molecular testing of all CRCs reveal that up to 28% of Lynch Syndrome patients could be missed with the most liberal clinical criteria, the Revised Bethesda Guidelines [8]. Furthermore, studies evaluating cost effectiveness of the various strategies have largely favoured universal testing, with initial IHC testing followed by* BRAF* mutation analysis for MLH1 absent tumours emerging as the most cost-effective approach [9].

As with any aspect in medicine, identification of these mutations must always be placed in the context of the patient and their respective wishes. A Dutch study revealed that almost half of the subjects in their cohort of family members at risk did not opt for genetic testing for LS [10]. Leenen et al. [11] further explored the motivations for the uptake or decline of genetic testing and revealed that the most important reasons for declining testing were anticipating problems with life insurance and mortgage, being content with life as is, and not experiencing any physical complaints.

Taken altogether, the present system of reliance on histories and patients to report their family histories appears to be inadequate and needs modification. A system such as that suggested by the latest AGA Guidelines where all cancers are universally tested appears to offer a cost-effective solution to these problems; however, it is essential that these results are interpreted in the perspective of the patient and the potential impact on their respective livelihood.

## Figures and Tables

**Table 1:** Amsterdam II, Revised Bethesda Guidelines, and HCP Referral Criteria.

*Amsterdam Criteria II*

Requiring three or more relatives with Lynch-associated cancer^*∗*^ in addition to the following
(1) One affected patient should be a first-degree relative of the other two
(2) Two or more successive generations are affected
(3) One or more affected relatives received diagnosis at age younger than 50 years
(4) FAP is excluded. Tumours should be verified by pathological examination
^*∗*^ *Endometrial, stomach, ovarian, pancreas, ureter, and renal pelvis *

*Revised Bethesda Guidelines*

(1) CRC diagnosed in a patient who is younger than 50 years of age
(2) Presence of synchronous, metachronous CRC, or other Lynch-associated tumours
(3) CRC diagnosed in a patient who is younger than 60 years of age, with the presence of tumour infiltrating lymphocytes, Crohn's-like lymphocytic reaction, mucinous/signet-ring differentiation, or medullary growth pattern
(4) CRC diagnosed in a patient with one or more first-degree relatives with a Lynch-associated tumour, with at least one of the cancers being diagnosed at age younger than 50 years
(5) CRC diagnosed in a patient with two or more first- or second-degree relatives with Lynch-associated tumours, regardless of age

*HCP Criteria* [6]

Any of the following
(1) Personal history of CRC diagnosed at age ≤ 40
(2) Personal history of Lynch syndrome related cancer at any age with IHC-deficient/MSI-H results
(3) Personal history of 2 Lynch syndrome related cancer diagnoses, including at least 1 colorectal cancer and a cancer diagnosed at age ≤ 50
(4) Family history that includes
(a) a close relative with personal history as above OR
(b) 2 first-degree relatives with a Lynch syndrome related cancer, both diagnosed at age ≤ 50 and including at least 1 diagnosis of CRC OR
(c) 3 or more Lynch syndrome related cancers, involving more than 1 generation, at least 1 case of CRC, and at least 1 case diagnosed at age ≤ 50.

**Table 2:** Summary of data from chart reviews.

*Consultations*	*N*
Consultations available	242
Age of diagnosis	242
≥60	174
50–59	48
40–49	18
30–39	1
<30	1
Past medical hx	242
Positive hx of any malignancy	39
Negative hx of any malignancy	203
Family hx	235
Positive family hx of malignancy	109
Negative family hx of malignancy	83
Negative family hx of GI malignancy	43
Full consultations not available	4
Total consultations attempted for review	246

*Pathology reports*	*N*
Pathology reports available	244
Age ≥ 60	176
Age < 60	66
Tumour infiltrating lymphocytes	3
Crohn's-like lymphocytic reaction	0
Mucinous/signet ring differentiation	1
Medullary growth pattern	0
Other	62
Full pathology report unavailable	2
Total pathology reports attempted for review	246

*Colonoscopy and OR notes*	*N*
Colonoscopy and/or OR notes available	246
Synchronous lesions	7
Metachronous lesions	7
Neither synchronous or metachronous	228
Full colonoscopy and/or OR notes unavailable	0
Total colonoscopy and OR notes attempted for review	246

**Table 3:** Characteristics of patients who received further investigation compared to all patients.

	All patients	Patients receiving further investigation	*p* value
	(*n* = 246)	(*n* = 13)	
*History*			
Average age of diagnosis	66	49	**<0.01**
Proportion of patients with past medical hx of malignancy	0.16	0.38	**0.03**
Proportion of patients with positive family hx of malignancy	0.46	0.92	**<0.01**

*Pathology*			
Proportion of patients with age < 60 and high risk pathology^*∗*^	0.016	0.077	0.08

*Colonoscopy & OR findings*			
Presence of metachronous or synchronous lesion	0.057	0.15	0.132

^*∗*^High risk pathology defined as any of the pathology findings fulfilling the Revised Bethesda Guidelines.
